# The New York Institute of Stomatology

**Published:** 1905-01

**Authors:** 

**Affiliations:** The New York Institute of Stomatology


### Reports of Society Meetings.

### THE NEW YORK INSTITUTE OF STOMATOLOGY.

A meeting of the Institute was held at the "Chelsea," No. 222 West Twenty-third Street, New York, on Tuesday evening October 4, 1904, the President, Dr. Brockway, in the chair.

The minutes of the last meeting were read and approved.

The President then introduced the essayist of the evening, Dr. N. S. Jenkins, of Dresden, Germany, who read a paper on "A New Porcelain for Prosthetic Purposes."

(For Dr. Jenkins's paper, see page 22.)

DISCUSSION.

*Dr. F. T. Van Woert.*—It would be hard to express gratitude at being present this evening and at the demonstration this afternoon. I personally feel under a deep obligation to Dr. Jenkins for his work in this line.

In the early days I was very sceptical as to the practical value of porcelain as a filling-material, and to the present just as sceptical regarding porcelain for bridge-work. But after seeing the demonstration this afternoon I am convinced that I shall have to take the matter up, as there seems a bright prospect for its success.

In the past I have considered porcelain bridges impracticable, on account of the ease with which they are broken. Dr. Jenkins assures me that with his new porcelain there is no such danger. This being so, it eliminates one of the most serious objections. I have thought of late that they might be practicable if set with the Evans cement, which I have found most admirable, in gold bridge cases.

In cases of a break-down, with this cement the bridge can be easily removed, and this, in consideration with Dr. Jenkins's new product, may make porcelain bridges practical.

I cannot see wherein there is very much to say except in commendation of Dr. Jenkins's work. I do not see any chance for a discussion, and I cannot find any fault.

The inlay material introduced by Dr. Jenkins is a very excellent one for adapting crowns to the roots. It will stand grinding and polishing and leave as smooth a surface as when first fused.

*Dr. C. F. Allan.*—Dr. Jenkins, with his years of laboratory experiments, pursued with the most patient care and at great personal sacrifice, is an apt illustration of the idea expressed so well in his paper, that "dentists themselves are the best judges of dentists' needs." The first sugestion of inlay material came to the essayist doubtless from his desire to do away with the unsightly display of gold in the mouth, his needs at first being only in the line of æsthetics and the imitation of nature; but he has builded so well that besides accomplishing this great purpose he has given us a material of the very first quality from the point of view of tooth preservation, and especially is it useful for those cases of large proximal and occluso fillings in the back teeth where much contouring is necessary and which could only be done in an inferior manner with gold at an enormous expenditure of time and labor on the part of the dentist and great suffering and nervous strain to the patient. Now, Dr. Jenkins has supplemented this great boon by adding this new material, of which he speaks to-day. He tells us that it is much stronger than the porcelain teeth we use, and will bear a heavier strain than the porcelain bodies.

The flowing of the material as demonstrated this afternoon is all that could be desired; it flows easily, stays put as you want it, and is tough and homogeneous.

It has been the opprobrium of our profession, especially in this country, that we have not imitated nature. All praise now to Dr. Jenkins, who has given us the means for holding the mirror up to nature; and the opprobrium in the future will be the greater if we do not take advantage of such means.

*Dr. H. L. Wheeler.*—It has given me much pleasure to listen to Dr. Jenkins this evening, and I think he deserves credit for so persistently advocating the æsthetic in dentistry. His constant endeavors can hardly fail to have a beneficial effect in persuading members of our profession to try to imitate nature more closely, and so minister to the beautiful; and as a man I admire him.

Upon reading this paper over several times, in the hope of finding some scientific statement that would admit of discussion, I have been unable to do so. In the first place this is an unsupported statement of an interested party and owner of a secret formula advocating this article as superior to everything else just as every man has done since commerce began. Under the circumstances it is difficult to discuss this paper as scientific papers usually are discussed. But there are some statements in this plea for the use of prosthetic porcelain to which I wish to call attention. For instance, in the statement that "materials employed in making dental porcelain have hitherto been worked somewhat crudely," I suppose he means porcelain used for dental purposes. There might be some difference of opinion concerning this if some of the other manufacturers were heard from. It is doubtful in my mind if prosthetic porcelain, so called, is a porcelain at all. I submitted a sample of porcelain enamel, which fuses at about 200° F. below prosthetic enamel, to one of our most distinguished and possibly the greatest authority on porcelain in this country, and he pronounced it glass. I have tried strenuously to get samples of this material of Dr. Jenkins's to experiment with, but find it is not obtainable. Its fusing-point, 1075° C., which, converted into Fahrenheit according to the essayist's statement, is from 1678° to 1768°, which is nearly or quite 200° below the lowest of the highest fusing bodies, would indicate that the fusing-point of the silicates had been considerably lowered by the addition of artificial means, or the doctor is using a silicate, that is, a feldspar containing an excessive amount of potash or soda, or some other alkaline material to lower the fusing-point, or he could, by breaking up and finely pulverizing and rebaking a number of times, secure similar results. In either case the results would not vary greatly. It is not impossible that his statement of its great strength is erroneous, for I am informed by men of ability, whose work is what might be called mineralogical chemistry, that either of the above-mentioned processes decreases the strength of the material. The statement that it can be used safely at a thickness of three millimetres, which, transposed, is about one-eighth of an inch, does not indicate that it has great strength. The inner cusps of bicuspid or artificial teeth are ground much thinner than this and last for years.

As to the question of the gold volatilizing, it is a matter of indifference if it does. I have put pieces of platinum together, using pure gold as a solder and run the heat up to 2600° F. The gold has apparently disappeared, but the pieces of platinum were held together as though they were one piece. The statement that the fusing-point of gold is 1075° C. (1903° F.) is, according to such authorities as I have been able to find, 113° out of the way. They give the fusing-point of pure gold as about 2016° F. Now, our essayist may make mistakes, as this shows, and it would not be the first time a good man had been in error, and it seems to me that it might be well to be cautious in the use of this material, at least until it shall have time to prove its merits, if it have any.

That the material may prove to be useful does not in my estimation warrant its unlimited use until its lasting qualities are better known. Although proprietary compounds may be very useful and of great benefit, the burden of proof is upon those who advocate their use. In regard to iridio-platinum for full upper plates, I have known of this being used in artificial dentures, and although there is great difficulty in swaging it, it can be used successfully where a vulcanite attachment is used. But as for heating it, in order to solder artificial teeth to it, the evidence thus far seems to be that it will spring badly and ruin the fit; hence I doubt its practicability.

*Dr. Houghten.*—I would like to ask Dr. Jenkins what the percentage of shrinkage of his porcelain is?

*Dr. Jenkins.*—Every porcelain has a certain amount of shrinkage if it is really fused. You can take some so-called porcelains and subject them to a very high degree of heat without fusing them; indeed, there are so-called porcelains that no amount of heat will thoroughly fuse. It is always a question of the way in which the materials have been treated. In order to have a porcelain adapted for practical use, it must fuse to a very great extent, and such a porcelain will shrink. The percentage of shrinkage of this porcelain is very similar to that of the inlay porcelain or of any porcelain that is thoroughly fused. However, this is a thing that is of no great consequence in the practical working of the porcelain. Shrinkage is so uniform in any one grade of work in ceramics that no very great attention is paid to it after the first experiments with any particular body are made. It makes some difference as to how moist the piece is. In this material I am presenting to you the degree of shrinkage has never been measured. It is not really a practical question.

*Dr. George S. Allan.*—I should like to hear a little more definitely from Dr. Jenkins in regard to this iridio-platinum that was negatived by Dr. Wheeler. Dr. Jenkins says that iridio-platinum with ten per cent. iridium, is essential in work with this prosthetic porcelain. As I understand it, he has had two years' actual practice with this metal. The more or less constant use of a material is pretty conclusive as to its merits. When Dr. Jenkins first made the statement to me that he used platinum with such a high percentage of iridium in alloy, the question naturally came to my mind as to how this could be swaged. Dr. Jenkins tells me that it is not so difficult a matter. He uses Melotte's metal dyes. He did not allude to this springing quality mentioned by Dr. Wheeler. All of those present at the demonstration at my office were highly delighted with the simple and easy way in which this porcelain could be manipulated. I know very little about ceramics, and so do not pretend to criticise, but I look upon Dr. Jenkins's two years' use of this material in combination with the platino-iridium base as practically settling the question of its usefulness.

*Dr. Gaylord.*—I simply wish to add my personal experience with this prosthetic porcelain to what has already been said in favor of it. It is one thing to listen to remarks upon a subject, and another to experiment with your own hands. If any of you will procure some of this material and try it in your own laboratory, you will realize to your own satisfaction its great strength and its perfect adaptation to whatever it comes in contact with. The matter of shrinkage seems to me to be of very little concern. Its great strength and the character of work it will enable us to do is highly gratifying. I believe Dr. Jenkins is too well known to bring forward anything that we cannot endorse. If he indorses it, it is sufficient for me.

*Dr. George S. Allan.*—Just one more point. All bodies that are used for artificial teeth and continuous gum work are hard and brittle. This body, as I understand Dr. Jenkins, is hard and tough and not as easily broken as former bodies.

*Dr. Stockton.*—I have always been a kind of hero-worshipper. I like a man who leads. I think that is why I have always been such an admirer of Napoleon. That is why I admire Dr. Jenkins.

I had the pleasure of being at the dental clinics at St. Louis, and the centre of attraction of these clinics was the inlay work. The appearance of this work alone is enough to attract us to it. The only question that comes up with this and all porcelain work is, "Is it strong enough to last?" Whatever Dr. Jenkins tells us we can rely upon, and he tells us that it is strong enough to stand the strain for which it is intended. The interest is so great in inlay work that our humble society in New Jersey, the Central Dental Association, is to have the first clinic it has ever devoted entirely to this work.

*Dr. Palmer.*—I cannot let the occasion go by without speaking of this work. Fourteen-odd years ago I was forced to take up a different line of bridge-work because of difficulties with the International Tooth Crown Company. Since that time I have used various bodies for bridge-work, but none which were satisfactory. Some few months ago my attention was called to this porcelain of Dr. Jenkins's, and I succeeded in getting some of it. I have used it in a good many small pieces, and have found it excellent, even using plain rubber teeth without having any trouble whatever. With it

I have had so much better results than with anything else that I am delighted. With all due respect to Dr. Wheeler, it does not make any difference to me whether it contains any glass or not; it looks better than anything I have ever used, and I believe it is going to wear better.

*Dr. C. F. Allan.*—Dr. Jenkins recommends using pure gold for solder. There is nothing better. I have had some experience with platinum gold solder, and it has been very unsatisfactory. It does not flow in the beautiful manner that gold does, and does not begin to be as strong, and as the strength of the framework is essential in the use of porcelain for bridge purposes, I would give this word of warning.

*Dr. Jenkins.*—I wish to thank Dr. Wheeler for his kind criticism. If he has interested himself in ceramics he must know that the materials of which all the finer porcelains are made are always essentially the same,—silica, feldspar, and kaolin. It is only a question as to how they are prepared, in what proportions they are compounded, and how they are fluxed. The production of a high-fusing porcelain is an exceedingly simple matter. As ordinarily produced, the high-fusing porcelains are but the threshold of a perfect material. There goes into this porcelain that I have prepared for prosthetic work at least twenty times as much work as is necessary to produce the ordinary high-fusing body. It is so complicated a process that if I were to take Dr. Wheeler into my laboratory and give him the materials and set him to work, I venture to say that it would take him two years before he could learn just when and how to unite the different ingredients. These things can be brought out only by painstaking and complicated experiments. In producing your results in this work you must simply pay no attention to time or expense. It is not only a question of the proportion of the ingredients, but of the different degrees of fineness as well.

Referring to the melting-point of gold, I beg leave to say that no one in all the world has yet been able to say what the absolute melting-point is. I believe that the Germans are conceded to be authorities on this subject, and in Germany it is stated to be 1075° C. There have been within the last few years a series of experiments made in England, and it has been announced there that the melting-point of gold must be conceded to be between 1064° and

1065° C. But the balance of authority I may say is with the German chemists who state it at 1075° C.

What I have endeavored to produce is a material that the ordinary man can use. When I spoke of high-fusing porcelain I did not mean to imply that it is crude, and of course it is a porcelain indispensable in certain forms of work, as, for instance, in the manufacture of porcelain teeth where an indestructible form is necessary. The requirements for a prosthetic porcelain are essentially different. It must be a porcelain which, when fused, will flow into the minutest crevice, which shall perfectly attach itself to the porcelain tooth around which it is placed so that it cannot be separated with violence. You can break away the tooth, but cannot break away the prosthetic porcelain. It is also of great importance that it possess great tenacity. There is an enormous difference too between the kind of glaze a porcelain takes, whether it is produced as a natural result of the fusing of the material or whether it is an enamel put on after the piece is practically finished. The prosthetic porcelain is of one consistency throughout, which makes it exceedingly enduring. You have all seen pieces of continuous gum baked after the old method, where the piece was first biscuited and then covered with a layer of enamel, and where after a time the enamel had worn off leaving the more porous porcelain exposed, and which in time became offensive. This cannot occur with the prosthetic porcelain, because it is the same throughout.

There were so many things mentioned, and there are so many things about it that I could tell you did time permit, that I shall have to ask you to take it upon faith. I can only tell you that I believe you will find it an indispensable adjunct to your practice, and that by means of it you will be able to eliminate any appearance of artificiality from the mouth. Even a clasp can be so completely covered up by this material that it becomes invisible. Of course, it must be a rigid clasp.

One word regarding platino-iridium. There is no question at all but that a properly alloyed platino-iridium can be swaged up. If one wished to strike up a cap, a band or half band, or a plate, either full or partial, ten per cent. platino-iridium can certainly be worked, perhaps not quite so readily, but when accomplished it is stiff and does not change with soldering or repeated meltings without any investment. In these cases there is no union between the alloy and the porcelain. Only perfect adaptation. Perhaps an attachment might be made by roughening the surface of the platinum, but it is seldom necessary.

I am very much obliged, gentlemen, for the patience with which you have listened to me.

Upon motion of Dr. F. Milton Smith, the Institute expressed to Dr. Jenkins its gratitude for his valuable discussion and demonstrations.

Adjourned.

FRED. L. BOGUE, M.D., D.D.S.,
*Editor The New York Institute of Stomatology.*

